# Zirconium Carboxyaminophosphonate Nanosheets as Support for Ag Nanoparticles

**DOI:** 10.3390/ma12193185

**Published:** 2019-09-28

**Authors:** Morena Nocchetti, Anna Donnadio, Eleonora Vischini, Tamara Posati, Stefano Ravaioli, Carla Renata Arciola, Davide Campoccia, Riccardo Vivani

**Affiliations:** 1 Department of Pharmaceutical Sciences, University of Perugia, Via del Liceo, 1, 06123 Perugia, Italyeleonoravischini@yahoo.it (E.V.); riccardo.vivani@unipg.it (R.V.); 2 Institute of Organic Synthesis and Photoreactivity, National Research Council, via P. Gobetti 101, 40129 Bologna, Italy; tamara.posati@isof.cnr.it; 3 Laboratorio di Patologia delle Infezioni Associate all’Impianto, IRCCS Istituto Ortopedico Rizzoli, via di Barbiano 1/10, 40136 Bologna, Italy; stefano.ravaioli@ior.it (S.R.); davide.campoccia@ior.it (D.C.); 4 Department of Experimental, Diagnostic and Specialty Medicine, University of Bologna, via San Giacomo 14, 40126 Bologna, Italy

**Keywords:** zirconium phosphonates, nanosheets, silver nanoparticles, antibacterial

## Abstract

A layered insoluble inorganic-organic solid, namely zirconium phosphate glycine-N,N-bismethylphosphonate, was used to prepare dispersions of nanosheets to support active metals such as metallic silver nanoparticles and zinc ions. Zr phosphate-phosphonate microcrystals were first exfoliated with methylamine to produce a stable colloidal dispersion and then the methylamine was removed by treatment with hydrochloric acid. The obtained colloidal dispersion of Zr phosphate-phosphonate nanosheets was used to immobilize silver or zinc cations, via ion exchange, with the acidic protons of the sheets. The layered matrix showed a great affinity for the metal cations up taking all the added cations. The treatment of the dispersions containing silver ions with ethanol yielded metal silver nanoparticles grafted on the surface of the layered host. The samples were characterized by X-ray powder diffraction, elemental analysis transmission electron microscopy, and selected samples were submitted to antimicrobial tests. The nanocomposites based on Ag nanoparticles showed good bactericidal properties against the bacterial reference strain *Staphylococcus epidermidis* (*S. epidermidis*).

## 1. Introduction

This period has been defined as “the antibacterial resistance era” in which the treatment of bacterial infection through the use of antibiotics can no longer be guaranteed. The massive use of antibiotics in human and animal diseases, cattle-breeding, and agriculture has led to the emergence and spread of drug-resistant bacterial strains. Indeed, microorganisms constantly evolve and are able to efficiently adapt to new environments [1].

Antibiotic resistance of bacteria can be experienced in biofilm mode, the formation of a layer of polysaccharides, proteins, and eDNA in which bacteria encase themselves [2]. The formation of biofilm on the surface of implanted medical devices is correlated with the incidence of persistent infections and with their inherent resistance to antibiotic chemotherapy [3]. Strategies for prevention of medical devices infections have been especially directed to reduce the vulnerability of the surfaces to bacterial adherence, colonization, and biofilm formation [4,5,6,7,8,9,10]. In this context, chemical and biological functionalization of devices represents a more recent approach to surface modification that involves either use of drug delivery systems or biomolecule grafting. Moreover, anti-infective properties have been conferred to implants by immobilizing on their surface active metals, nitric oxide (NO)-releasing materials, or non-antibiotic antibacterial substances in order to avoid the development of multiresistant bacterial strains [11]. Nanotechnology is opening up new frontiers in the design of anti-infective surfaces [12,13,14,15]. The application of metals and metal oxides, such as Ag, Au and ZnO, in the form of nanometric-sized particles as antimicrobial coatings is steadily increasing due to a series of distinctive features of such nanomaterials, including a high surface area and chemical reactivity, which allows high bioactivity, even in small doses [16]. Specifically, Ag nanoparticles are widely used as antimicrobial agents and their activity has been shown to depend on their shape and size [17,18,19]. The biocidal action of Ag nanoparticles has been correlated to various mechanisms, one of which is the release of their ions in solution [20,21,22] with consequent production of reactive oxygen species (ROS) and damage to proteins and membranes. Similar antimicrobial activity has been reported for nanostructured ZnO and for zinc complexes with the quinolone, which can generate a high local concentration of Zn^2+^, with detrimental effects on cell growth and tissue formation, and with the induction of cell apoptosis due to ROS formation [23,24,25].

In this context nanoparticles can act as ion reservoirs, which can progressively release them while maintaining a high activity and a low ion concentration for several days. The biocidal activity of metal nanoparticles is also related to other mechanisms that depend on their size; for example, they are able to adhere to the bacterial cell membrane causing holes and defects through which they can subsequently penetrate inside the cell.

However, some side effects may occur because of a large release of inorganic nanoparticles or metal ions that can cause toxicity and induce cytotoxicity [26]. To overcome this problem, metal nanoparticles can be immobilized on solid supports in order to decrease the exposure of the organism to high doses of nanoparticles and confine the release of metal ions to limited areas. Immobilization of metal nanoparticles can also avoid the aggregation of nanoparticles, a phenomenon that otherwise occurs easily and that leads to a decrease in their surface area and reduction of antimicrobial activity. [27,28]. Among solid support, zirconium phosphates and phosphonates have been intensively studied as supports for bioactive species, due to their peculiar properties: generally, they are highly insoluble, well tolerated by the organism, and have a modular structure that can be easily adapted to improve affinity toward selected guest species [29]. Recently our research group synthetized the zirconium glycine-N,N-bismethylphosphonate phosphate (ZG), an insoluble layered solid with formula: Zr_2_(PO_4_)H_3_(L)_2_ H_2_O [L = (O_3_PCH_2_)_2_NHCH_2_COO] [30]. The presence of the carboxyl and amino groups confer to the solid coordinative capabilities enhancing the affinity of this material towards transition metals [31,32,33,34]. Besides good ion exchange capacities, ZG is endowed with excellent intercalation properties, that is, the insertion of polar host species within the interlayer region [35]. Generally, the species intercalated in layered solids tend to organize themselves in the interlayer region forming true crystalline intercalation compounds, characterized by expanded interlayer regions. This property allows the use layered materials as reservoirs of molecular species, which are then protected from external agents, and which can then be released following a specific chemical signal [36]. However, a peculiar and unusual behaviour for this type of materials has been recently described for this compound, that is its complete exfoliation after intercalation of suitable molecules, for example n-propylamine [31]. The insertion of such species in the interlayer region causes the increase in the distance between the layers, hence the decrease of their interaction, that is not accompanied by equally strong interactions between layers and guest species. Finally, the solvation of layers induces a complete exfoliation of lamellae with the formation of a colloidal dispersion of single separated layers. However, the individual lamellas do not undergo structural changes and maintain their integrity. The thickness of the dispersed lamellae was evaluated using an atomic force microscope and was found to be around 3 nm, which corresponds to the stacking of about two or three individual layers. This phenomenon greatly increases the accessibility of the functional groups placed on the layer surface.

Aim of this study is the preparation of highly dispersed materials constituted of Ag nanoparticles (Ag NPs) or Zn ions efficiently anchored on ZG. Ag NPs, with size in the range of a few nanometers, have been generated in situ on the surface of exfoliated ZG layers resulting in colloidal Ag@ZG nanocomposites. Drying of these colloidal dispersions on a solid surface produces its homogeneous covering with the functionalized ZG layers. In order to test the antimicrobial activity of these systems, we have prepared small paper discs covered with the above mentioned materials.

## 2. Materials and Methods

### 2.1. Materials

ZrOCl_2_·8H_2_O was a Pro Analysi product (Merck, Milan, Italy). N,N-bis(phosphonomethyl)-glycine, phosphoric acid, hydrofluoric acid, AgCH_3_COO, Zn(CH_3_COO)_2_, methylamine and Minimum Essential Medium (MEM) were purchased from Sigma-Aldrich (Milan, Italy). All reagents were used as received without further purification.

### 2.2. Synthesis of Zr_2_(PO_4_)H_3_(L)_2_ H_2_O [L = (O_3_PCH_2_)_2_NHCH_2_COO] (ZG)

The synthesis of ZG was achieved as reported previously [20]. Briefly, N,N-bis(phosphono-methyl)glycine (L, 2.37 g, 9 mmol) was dissolved in deionized water (93 mL, 0.097 M), and then 1 M phosphoric acid (6 mL) was added to this solution. ZrOCl_2_·8H_2_O (1.93 g, 6 mmol) was dissolved in 2.9 M HF (20.4 mL, 59 mmol, molar ratio HF/Zr^IV^ = 10). These two solutions were mixed in a 500 mL Teflon bottle and placed in an oven at 90 °C. After three days the solid was filtered under vacuum, washed three times with deionized water, and dried at 60 °C for 24 h. The empirical formula was Zr_2_P_5_O_21_C_8_N_2_H_19_ (FW = 816, ion exchange capacity, IEC, = 3.67 mmol/g).

### 2.3. Preparation of Exfoliated and Regenerated ZG

ZG (300 mg, 0.367 mmol) was suspended in deionized water (29 mL) and then methylamine (11 mL, 0.1 M, 1.1 mmol), corresponding to 100% of ZG IEC, were added under vigorous magnetic agitation and the dispersion was kept under stirring at room temperature for 24 h.

The dispersion was equilibrated with 20 mL of 1 M hydrochloric acid for two h. The gel, recovered by centrifugation (13000 rpm, 10 min), was washed two times with de-ionized water and then dispersed in 30 mL of deionized water, obtaining a stable colloidal dispersion of exfoliated ZG (ZG-e). The content of dry solid in ZG-e, determined by drying measured volumes of the dispersion in oven at 100 °C, was 9.1 mg/mL.

### 2.4. Preparation of Ag@ZG and Zn/ZG

Several samples containing increasing amounts of silver were prepared starting from ZG-e. 16.7, 8.35, 4.2 and 2.1 mL of aqueous 0.06 M AgCH_3_COO were added dropwise under vigorous stirring, to appropriate volumes of ZG-e containing 273 mg of ZG, in order to have exchange rates equal to 100%, 50%, 25% and 12.5% of its IEC, respectively. The samples were identified as Ag@ZG-x, with x = 1, 0.5, 0.25 and 0.125 to consider the calculated exchange. The dispersions were left under magnetic stirring for 1 day. The solids were recovered by centrifugation (15000 rpm for 10 min) and washed twice with water. The samples were equilibrated for 12 h at room temperature in 70 mL of ethanol. Finally, they were recovered by centrifugation, washed twice with water and dispersed in 15 mL of de-ionized water. ZG exchanged with zinc cations (Zn/ZG) was prepared following the procedure described above except for the reduction step. Briefly, aqueous 0.25 M Zn(CH_3_COO)_2_ (1.9 mL) was added to a ZG-e volume containing 250 mg of ZG in order to have an exchange equal to 100% of IEC; the mixture was left under stirring for 24 h. After washing with de-ionized water, the recovered gel was dispersed in 15 mL of de-ionized water.

### 2.5. Silver Release Test

Silver release studies of Ag@ZG-0.125, was performed in Minimum Essential Medium (MEM) cell culture media at 37 °C. 8 mL of Ag@ZG-0.125 dispersion (containing 14.4 mg of solid/mL) were dispersed in 50 mL of MEM and were kept under stirring in bath thermostated at 37 °C. Aliquots (2 mL) of the acceptor fluid (MEM) were collected at predetermined time intervals and immediately replaced by the same volume of MEM equilibrated at 37 °C. The withdrawal MEM was diluted to 10 mL with 5 M HNO_3_ and the amount of silver released was detected by Inductively Coupled Plasma-Optical Emission Spectrometers (ICP). The results were normalized on the basis of the total silver content.

### 2.6. Characterization of Antibacterial Properties

#### 2.6.1. Sample Preparation

The bactericidal properties of the test materials were investigated by agar diffusion assay, using filter paper disks loaded with dispersions and measuring the halos of inhibition of bacterial growth around disks. For the preparation of the specimens, drops of the dispersions of Ag@ZG-0.125 (containing 14.4 mg of solid/mL), Zn/ZG (containing 15.3 mg of solid/mL) and ZG-e (containing 9.1 mg of solid/mL) were deposited on both sides of paper disks having a diameter of 10 mm. Then, the loaded disks were dried at room temperature. The amount of the solid deposited was of about 2.7 (±0.2) mg per disk.

#### 2.6.2. Bacterial Cultures and Agar Diffusion Assay

The tests were conducted on the bacterial reference strain *Staphylococcus epidermidis* RP62A (ATCC 35984). Fresh cultures of the bacterial strain were prepared from frozen stocks stored at −80 °C in the bio-bank of the ISO 9001:2008 certified Research Unit on Implant Infections of the IRCSS Istituto Ortopedico Rizzoli (Bologna, Italy). Bacteria were first plated on tryptic soy agar plates (TSA, cat. n. B19420, MEUS S.r.l., Piove di Sacco, Italy) at 37 °C and then cultured in Tryptose Broth (TB, cat. n. 552155, Biolife Italiana srl, Milan, Italy) at 37 °C until reaching an optical density of 0.1 at 625 nm (corresponding to a bacterial suspension of about 10^8^ CFU/mL). Mueller Hinton Agar (MH II Agar, cat. n. B19372, MEUS) plates were prepared by seeding and 3 to 4 disks for each treatment were deposited in the central part of the agar plate. Untreated paper disks and paper disks loaded with 50 U penicillin and 0.05 mg streptomycin (Penicillin-Streptomycin, cat. n. ECB 3001D, Euroclone, Pero, Italy) were used as negative and positive control, respectively. Agar plates were cultured for 24 h at 37 °C. Inhibition halos were measured and photos were taken of all agar plates. Overall, 10 disks were used for each condition and the diffusion assay was independently repeated 3 times.

#### 2.6.3. Bactericidal Activity by Direct Contact with Test Materials

In order to check bacterial growth/viability also in the area underneath the samples, the disks from agar diffusion experiments were transferred in a 24-well plate, frozen and stored at −80 °C overnight. Bacterial growth at the paper disk-agar medium interface was indirectly estimated by the amount of ATP. The BacTiter-Glo Microbial Cell Viability Assay from Promega Italia S.r.l. (cat. n. 48231, Milan, Italy) was adapted to quantify the ATP of bacteria adhered to the specimens. Briefly, 1 mL of sterile physiologic saline solution was added to each well containing a transferred disk (4 disks had been used for each treatment) and to two empty wells used to check the background. The substrate of the BacTiter-Glo assay was reconstituted in its buffer in dark conditions, following the indications provided by the producer of the kit. A volume of 1 mL of reconstituted substrate was added to each well. After 5 minutes of incubation at room temperature in the dark, duplicated aliquots of 100 µL were taken from each well and transferred in two wells of a black 96-well plate for bioluminescence reading by a Modulus II multifunction plate reader (Turner BioSystems, Sunnyvale, CA, USA). Bioluminescence readings were finally expressed as Log_10_ of relative luminescence units (RLU).

### 2.7. Instrumental Procedures

The samples were characterized by X-ray powder diffraction (XRPD), transmission electron microscopy (TEM), inductively coupled plasma - optical emission spectrometry (ICP-OES), and zeta-potential measurements.

XRPD patterns were recorded by a X’PERT PRO diffractometer (PANalytical, Royston, UK) operating at 40 kV and 40 mA, with a 0.030° 2θ step size, and 40 s step scan, using the Cu Kα radiation and an X’Celerator detector (PANalytical, Royston, UK).

The morphology of the samples was investigated with a model 208 transmission electron microscope (TEM, Philips, Eindhoven, Nederland), operating at an accelerating voltage of 100 kV. The samples in form of dispersions were deposited on a copper grid (200 mesh) precoated with a Formvar film and then evaporated in air at room temperature. Ag NP surface density was estimated by counting the number of Ag NPs in selected areas of TEM images. The density was expressed as number of Ag NPs μm^−2^.

Zr, P, Ag and Zn analyses were performed with a 700-ES series ICP-OES spectrometer (Varian, Santa Clara, CA, USA). Samples (5 mg) were dissolved in concentrated HF, then 2 mL of concentrated HNO_3_ and deionized water were added tol a final volume of 100 mL. The final solution was directly injected into the instrument.

The zeta-potential was measured at 25 °C in aqueous solutions (0.15 mg mL^−1^) was determined by photon correlation spectroscopy (PCS) at 25 °C using a NanoBrook Omni Particle Size Analyser (Brookhaven Instruments Corporation, Holtsville, NY, USA) equipped with a 35 mW red diode laser (nominal 640 nm wavelength).

## 3. Results and Discussion

### 3.1. Zirconium Phosphate Glycine-N,N-Bismethylphosphonate

For sake of clarity, here we recall the structural characteristics of the host compound. ZG is an insoluble layered solid, with formula: Zr_2_(PO_4_)H_3_(L)_2_ H_2_O [L = (O_3_PCH_2_)_2_NHCH_2_COO]. It can be easily prepared by direct synthesis by thermally decomposing Zr hydrofluoric complexes of ZrF_6_^2–^ type, in the presence of an adequate mixture of phosphoric acid and N,N-bisphosphonomethyl glycine [30].

**Figure 1:**
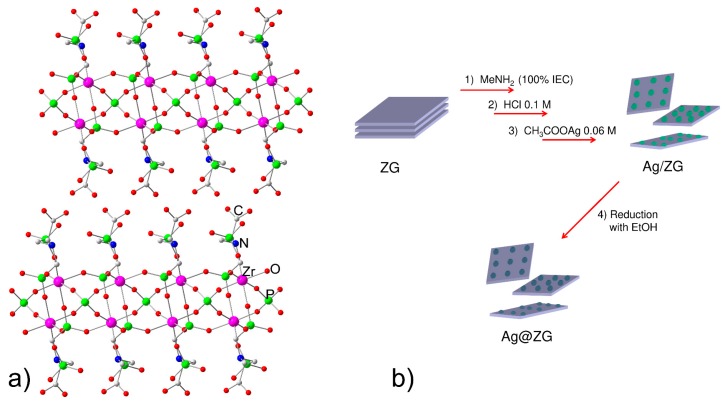
(**a**) Schematic representation of the structure of zirconium phosphate glycine-N,N-bismethylphosphonate (ZG); (**b**) Synthetic procedure used to prepare Ag@ZG.

Figure 1a shows a sketch of the structure of ZG. The compound crystallizes in the monoclinic system, space group C2/c. The structure of the compound is constituted by the stacking of covalent layers interacting each other only by hydrogen bonds.

The thickness of each layer is about 15 Å and the structure is characterized by ZrO_6_ octahedra linked, through the vertices, to tetrahedral groups of PO_4_ and PO_3_C, the latter belonging to the glycine diphosphonate. The PO_4_ phosphate groups are placed inside the layers and are tetradentate, while the glycine diphosphonate groups are positioned on the outside of the layers so that the surface of layers is rich of amino, P-OH, and -COOH groups. The packing of these building units creates a very polar interlayer region, with three acidic protons per unit formula, attributable to carboxyl and P-OH groups; these are easily exchangeable with other cations and give an ion exchange capacity of 3.67 milliequiv/g.

### 3.2. Preparation and Characterization of Ag@ZG and Zn/ZG

**Figure 2:**
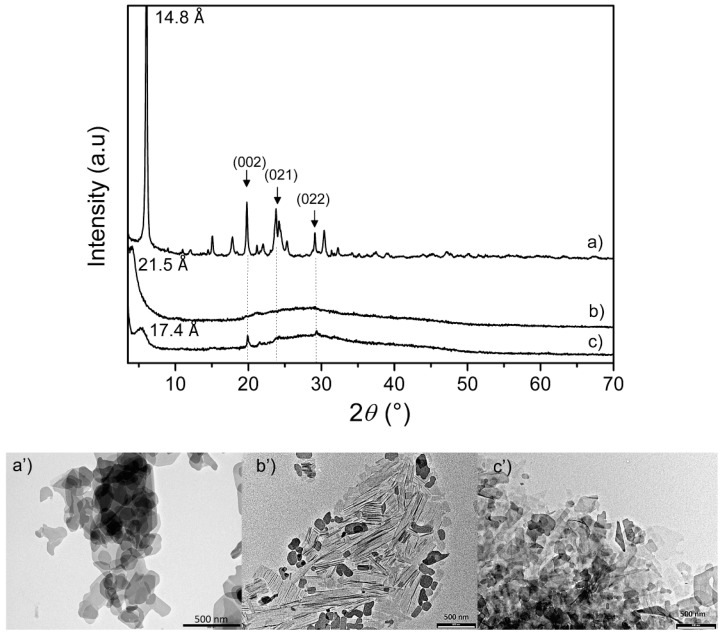
XRPD patterns and TEM images of microcrystalline ZG, (**a**,**a’**); centrifuged colloidal dispersion of ZG -MeNH_2_; (**b**,**b’**); centrifuged ZG-e; (**c**,**c’**). The numbers in brackets indicate the Miller indices related to the various reflections.

The synthetic procedure used to prepare Ag@ZG is summarized in Figure 1b. To increase the accessibility of the surface of layers, a sample of ZG suspended in water was first exfoliated by adding methylamine until 100% of its IEC. The intercalation of methylamine, that occurs via an acid-base reaction, provokes the breaking of hydrogen bonds connecting the layers and the exfoliation of the solid. Figure 2 shows the comparison between the XRPD pattern of crystalline ZG and the centrifuged gel obtained after delamination. In the gel most of the material is still delaminated, although a weak peak relative to the interlayer distance of the intercalation compound, about 21.5 Å, indicates that a small fraction of layers is re-aggregated following centrifugation. The interlayer distance of this is increased by about 7 Å from the initial value of 14.8 Å, due to the presence of the guest species and water molecules between the layers.

After that, the dispersed sample was treated with HCl in order to completely remove the amine, which could be toxic and competitive in the subsequent interactions with metal ions, and to obtain a dispersion of ZG sheets in which acidic P-OH and carboxylic groups present on the surface of layers are regenerated in their protonated form. This dispersion will be indicated as ZG-e in the following. After treatment with HCl the system was found to maintain most of its dispersed state, as assessed by XRPD analysis, reported in Figure 2c. The pattern of the centrifuged ZG-e shows a weak and broad peak at 17.4 Å that is likely related to a small fraction of re-aggregated layers containing a higher amount of hydration water. After complete drying of this sample at 80 °C, the formation of the original ZG phase, although less crystalline, was observed. The pattern of ZG-e also shows some very weak reflections that can be referred to atomic planes that depend on the internal structure of layers and not on their stacking. Indeed, these peaks correspond to those peaks of crystalline ZG, having no components along the crystallographic axis *a*, that is the axis perpendicular to the layers, and therefore with the Miller index *h* equal to zero. This observation can indicate that, although the crystals are destructured by exfoliation, the internal structure of the individual layers is preserved.

A zeta potential measurement of the aqueous dispersion of ZG-e (pH = 6.5) gave a highly negative value: −51 mV; this value is compatible with a fairly stable dispersion, as found by Saxena et al. for zirconium phosphate [37].

The morphology of the pristine ZG, after exfoliation, and after regeneration, was studied by TEM; selected images are reported in Figure 2. ZG is constituted by irregular platelets with diameter ranging from 1 μm to 100 nm (Figure 2a’), corrugate and very thin structures appear after exfoliation very likely ascribable to single layers (Figure 2b’) while the acidic treatment provokes a partial aggregation of the particles (Figure 2c’).

ZG-e was equilibrated with increasing volumes of 0.06 M AgCH_3_COO in order to exchange the ZG protons by silver cations. The recovered solids (namely Ag@ZG -x, with x = 0.125, 0.25, 0.5, 1, see experimental) were successively washed by a bland reducing agent as ethanol to form Ag NPs. The amount of silver added and uptaken per mol of zirconium is reported in Table 1, where, for the sake of clarity, the added silver is also given as percentage of ion-exchange capacity. Note that almost all the added silver is uptaken indicating, as already observed, a great affinity of the solid for the metal cations.

The efficient immobilization and wide dispersion of Ag NPs on the surface of nanosheets depends on the delamination step and the peculiar properties of ZG to exfoliate under appropriate conditions. The dispersions of Ag@ZG, after washing with ethanol, show a partial re-aggregation, as also confirmed by the measurement of zeta potential for the Ag@ZG-0.125 re-dispersed in water, which resulted −24 mV, considerably higher than that obtained for the dispersion of ZG-e.

**Figure 3:**
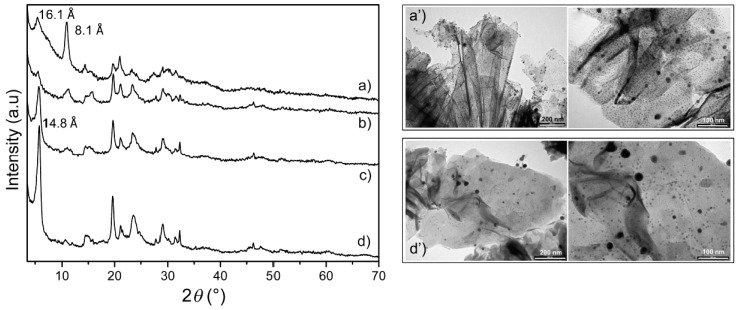
XRPD patterns and TEM images of Ag@ZG-1, (**a**,**a’**); Ag@ZG-0.5, (**b**); Ag@ZG-0.25, (**c**); and Ag@ZG-0.125, (**d**,**d’**).

XRPD patterns of dried dispersions of Ag@ZG are reported in Figure 3. Crystallinity of these samples is much lower than that of pristine ZG, probably because the repacking of sheets during the drying process is quite messy. However, the patterns of low exchanged samples (curves c and d) closely correspond to that of crystalline ZG, probably indicating that all silver ions left the interlayer region due to the reduction and NP formation process; on the contrary, the pattern of the 100% exchanged sample shows a higher interlayer distance, 16.1 Å as compared to 14.8 Å of pristine ZG, and a lower crystallinity, probably indicating the presence of intercalated silver cations. In addition, the increased intensity of the second peak, at 8.1 Å, a half of the interlayer distance, may indicate the formation of a high electron density region, parallel and comparable to that of the zirconium plane region, just at a half of the unit cell height, confirming the presence of residual silver ions in the interlayer region [38].

Independent of Ag loading, all patterns of Figure 3 still have in common the peaks related to the structure of layers and not to their packing, as discussed for Figure 2, indicating that, although defective packed, their structure still remains unchanged.

Despite the presence of a large number of Ag NPs, as observed by TEM (see later), no reflections related to metallic Ag were detected, suggesting that Ag NPs are very small, and their broadened peaks could not be distinguished from the background.

TEM measurements (Figure 3) were performed to determine the dimensions and the density of the Ag NPs on ZG. In all the samples the sheets appear covered by dark particles, ascribable to metallic silver and belonging to two different populations: one with dimensions larger than 10 nm, the other with dimension lower than 10 nm. Moreover, the density of Ag NPs decreases as the silver content decreases in the Ag@ZG samples. Indeed, the density of Ag NPs in Ag@ZG-1 was estimated to be 140 and 28600 Ag NP μm^−2^ for the larger and smaller particle populations, respectively; whereas the density of the Ag NPs in Ag@ZG-0.125 reduced to 70 and 3500 Ag NP μm^−2^ for the two populations. The mean dimensions of the smallest population are slightly affected by the silver loading ranging from 3.6 to 5.2 nm. Conversely, the mean dimensions of the larger population appear to decrease according with the silver loading reduction, from 36.3 nm to 16.7 nm for Ag@ZG-1 and Ag@ZG-0.125, respectively. In principle, the smallest particles and the lowest density of Ag NPs of this latter sample should provide a higher silver cation release and higher antimicrobial activity [39] and therefore it was selected as the best candidate for antimicrobial tests.

**Figure 4:**
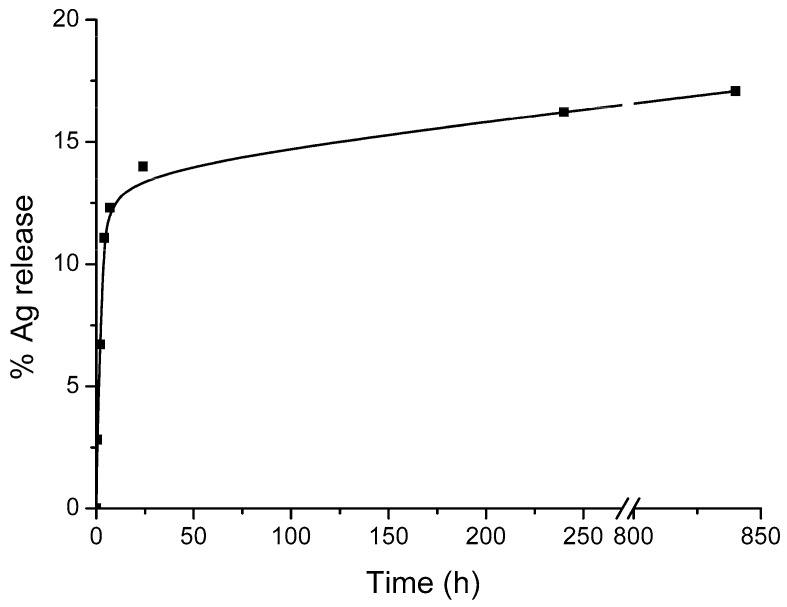
Silver release curve of Ag@ZG-0.125 in MEM cell culture media at 37 °C.

Figure 4 shows the total silver release from Ag@ZG-0.125 in Minimum Essential Medium (MEM) cell culture media. The release is characterized by an initial burst effect: the 12.3% of the silver is released in the first 7 h, after this the release becomes very slow and reached the 16% after 35 days.

**Figure 5:**
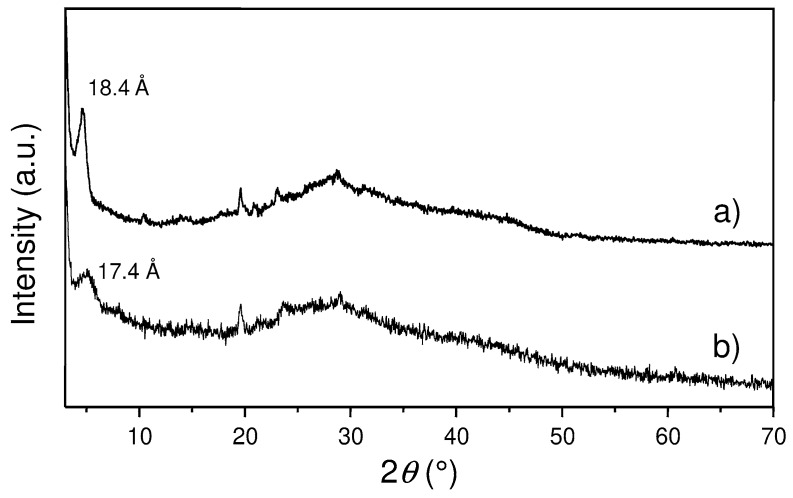
XRPD pattern of Zn/ZG (a); pristine ZG-e (b).

A sample containing zinc cations (Zn/ZG) was prepared equilibrating ZG-e with a solution of zinc acetate containing an equivalent zinc amount. The synthetic procedures followed the scheme reported in Figure 1b, except for the reduction step, that was not carried out. Elemental analysis showed that all the acidic protons were exchanged by zinc, confirming the affinity of this material towards metal cations. Comparison of XRPD patterns of the final product with that of the pristine ZG-e (Figure 5), revealed that Zn loading did not induce a significant recrystallization of the system, which maintained a high degree of dispersion.

### 3.3. Antibacterial Properties

**Figure 6:**
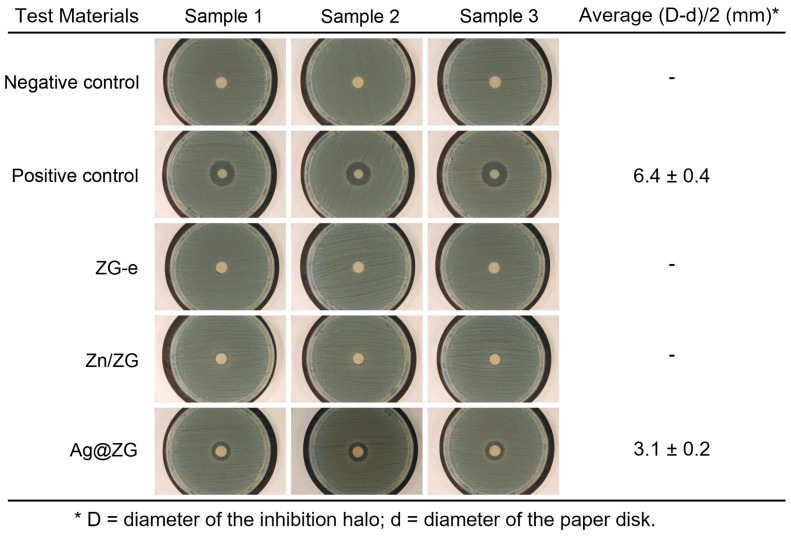
Agar diffusion assay: photos of the inhibition halos generated by the different treatments. These photos refer to one of the three independent experiments performed and are representative of the overall observations. Negative Control: untreated paper disk; Positive Control: paper disk loaded with 50 U penicillin and 0.05 mg streptomycin; ZG-e: ZG-e loaded paper disk; Zn/ZG: Zn/ZG loaded paper disk; Ag@ZG: Ag@ZG-0.125 loaded paper disk. The average sizes of the inhibition halos, and their standard deviations, are also reported.

The screening of the bactericidal properties of the test materials was conducted by the agar diffusion assay, through simple adaptations to enable the evaluation of the test materials that are in form of nanopowders. The results of the assay are reported in Figure 6 and show as Ag@ZG was the only test material that exhibited a bactericidal activity when challenged with the reference *S. epidermidis* strain RP62A. Indeed, neither ZG-e nor Zn/ZG showed circular inhibition halos around treated disks, while Ag@ZG constantly revealed a well distinguishable non-growth halo. As expected, the negative control did not exhibit any inhibition halo, while the positive control reproducibly displayed a halo extending over 6 mm from the margin of the disks.

The measurements of the inhibition halos obtained are also summarized in Figure 6. Even though the activity of Ag@ZG appears lower than that found for the combination of two antibiotics in the disk of the positive control, it has to be considered that the agar diffusion assay depends on the gradient of the leachable substances in the agar gel and cannot therefore be used for direct comparison. Given that the observable inhibition of bacterial growth extends well over 1 mm from the margin of the disk surface, it is possible to confirm that the material loaded with silver is endowed with evident bactericidal properties.

**Figure 7:**
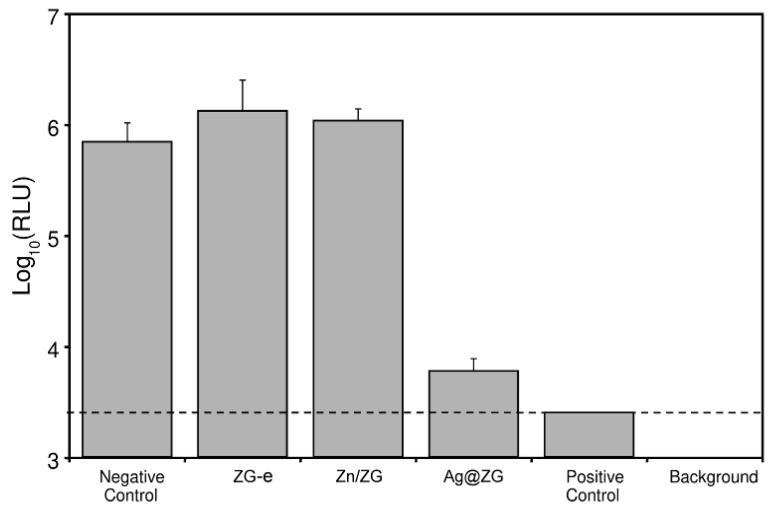
Bioluminescence measurements of the ATP detected in the disks of the different treatments. The bar graph reports the mean value ± S.D. of the measurements expressed as Log10 of relative luminescence values (RLU). For the negative control and the test materials, four different samples were measured. For the positive control the mean refers to two measured values.

Further, experimental work was conducted to ascertain whether the nanopowders of ZG e and Zn/ZG could have affected the bacterial growth by direct contact with the bacteria underneath the disks. A first attempt to assess the presence of bacteria on the bottom side of the disks was done by observation under fluorescence microscopy of the paper disks loaded with the two nanostructured materials after DNA staining with different fluorochromes. However, the intrinsic fluorescence of cellulose paper filter was found to interfere with the assessment and the identification of viable bacteria absorbed on the disk surface. For such reason, the measurement of ATP was thought to be a useful alternative approach to gain some indirect information on the presence of dead/viable bacteria. This second strategy proved to be successful. The sensitive measurements by bioluminescence were expressed as Log_10_ (RLU) and enabled to verify the level of retained bacterial ATP on the surfaces of control and treated paper disks (Figure 7). Underneath of ZG-e and Zn/ZG treated disks, it was possible to definitively identify the presence of abundant bacterial ATP to levels even slightly higher than the control untreated paper disks. These minor differences should probably be attributed to the varying absorption/adsorption of bacteria and free bacterial ATP from the bacterial loan formed on the agar surface to the disks. Indeed, the loading of the test materials on the filter could certainly be expected to result in little changes in the retention of bacteria and ATP when transferring the disks from the agar cultures. This said, through the bioluminescence measurements, it became clearly visible that, while for the Ag@ZG and even more for the positive control there was a drop of 2–3 logs, for ZG-e and Zn/ZG the presence of bacteria was considerable, with no sign of reduction with respect to the negative control. These findings would exclude that the latter materials could alter bacterial viability or even growth by direct contact.

It can be noticed that, even for the positive control, a substantial level of bioluminescence is detected. This implies that, after the exposure with seeded bacteria, the paper disks remain contaminated by residual bacterial ATP even if bacteria are probably killed and release their content. Marginal differences can be observed when comparing the negative control to ZG-e and Zn/ZG. The slight variations are likely related to the different retention of bacteria or of bacterial ATP, during the transfer from the agar plate. These findings would anyway exclude the hypothesis that Zn/ZG and ZG-e reduce bacterial viability by direct contact killing. Ag@ZG exhibits a drastic reduction of ATP with respect to the other test materials, corresponding to more than 2 Logs. The slight variation with respect to the positive control could again be attributed to a greater retention of the bacterial ATP released from the killing of the seeded bacterial cells as this assay has a high sensitivity, which can be estimated to be of the order of about 10^3^ CFUs.

## 4. Conclusions

In this work the peculiar property of ZG of giving rise to stable colloidal dispersions upon intercalation of polar molecules and its affinity towards transition metal cations, have been exploited to prepare colloidal dispersions of nanosheets functionalized with zinc ions or decorated with Ag NPs. Under the test conditions adopted, Ag@ZG showed good bactericidal properties against *S. epidermidis* while Zn/ZG and ZG-e alone had no appreciable antibacterial effect. These dispersions of functionalized ZG nanosheets, thanks to their ability to be deposited in thin layers, such as a varnish, can open up the possibility of modifying solid surfaces of various nature, and conferring them antimicrobial properties. Recent studies have highlighted the synergistic antimicrobial effect of silver when in combination with Zn^2+^ [40] or antibiotics [41]. It was found that silver administered in combination with antibiotics could resurrect ineffective antibiotics and render them effective against MRSA. Thus, future work aims at exploring the potential of multifunctional ZG-based materials obtained by loading different antibacterial substances combined in appropriate proportion.

## Figures and Tables

**Table 1:** Silver added and uptaken per mol of zirconium of ZG (IEC = 3.67 mequiv/g); weight percentage of silver in the Ag@ZG composites; Ag NPs dimensions measured by TEM.

Sample Ag@ZG -x	Ag^+^ Added mol/mol Zr (%IEC)	Ag^+^ Uptaken mol/mol Zr ^a^	Ag (w/w %) ^a^	Ag NPs Diameter (nm) ± SD ^b^
x = 0.125	0.19 (12.5)	0.18	4.6	16.7 ± 5 4.5 ± 2
x = 0.25	0.37 (25)	0.37	8.0	20. 3 ± 8 5.2 ± 2
x = 0.5	0.75 (50)	0.75	15.4	18.3 ± 6 3.8 ± 2
x = 1	1.50 (100)	1.50	26.7	36.3 ± 9 3.6 ± 2

^a^ Data obtained by ICP-OES analyses on the dissolved dried samples. ^b^ Average diameters are referred to two different populations of Ag NPs observed on TEM images; the values were calculated by measuring at least 100 NPs taken on several images.

## References

[1] Brown E.D., Wright G.D. (2016). Antibacterial Drug Discovery in the Resistance Era. Nature..

[2] Stewart P.S., Costerton J.W. (2001). Antibiotic resistance of bacteria in biofilms. Lancet..

[3] Høiby N., Bjarnsholt T., Givskov M., Molin S., Ciofu O. (2010). Antibiotic resistance of bacterial biofilms. Int. J. Antimicrob. Ag..

[4] Arciola C.R., Campoccia D., Speziale P., Montanaro L., Costerton J.W. (2012). Biofilm formation in *Staphylococcus* implant infections. A review of molecular mechanisms and implications for biofilm-resistant materials. Biomaterials.

[5] Arciola C.R., Caramazza R., Pizzoferrato A. (1994). In vitro adhesion of *Staphylococcus epidermidis* on heparin-surface-modified intraocular lenses. J. Cataract Refract. Surg..

[6] Tiller J.C., Liao C.J., Lewis K., Klibanov A.M. (2001). Designing surfaces that kill bacteria on contact. Proc. Natl. Acad. Sci. USA..

[7] Arciola C.R., Montanaro L., Moroni A., Giordano M., Pizzoferrato A., Donati M.E. (1999). Hydroxyapatite-coated orthopaedic screws as infection resistant materials: *in vitro* study. Biomaterials.

[8] Bazaka K., Jacob M.V., Crawford R.J., Ivanova E.P. (2012). Efficient surface modification of biomaterial to prevent biofilm formation and the attachment of microorganisms. Appl. Microbiol. Biotechnol..

[9] Wang G., Zreiqat H. (2010). Functional coatings or films for hard-tissue applications. Materials (Basel).

[10] Campoccia D., Montanaro L., Arciola C.R. (2013). A review of the biomaterials technologies for infection-resistant surfaces. Biomaterials.

[11] Parasuraman P., Antony A.P., B S.L.S., Sharan A., Siddhardha B., Kasinathan K., Bahkali N.A., Dawoud T.M.S., Syed A. (2019). Antimicrobial photodynamic activity of toluidine blue encapsulated in mesoporous silica nanoparticles against *Pseudomonas aeruginosa* and *Staphylococcus aureus*. Biofouling.

[12] Shah S., Gaikwad S., Nagar S., Kulshrestha S., Vaidya V., Nawani N., Pawar S. (2019). Biofilm inhibition and anti-quorum sensing activity of phytosynthesized silver nanoparticles against the nosocomial pathogen *Pseudomonas aeruginosa*. Biofouling.

[13] Raie D.S., Mhatre E., El-Desouki D.S., Labena A., El-Ghannam G., Farahat L.A., Youssef T., Fritzsche W., Kovács Á.T. (2018). Effect of novel quercetin titanium dioxide-decorated multi-walled carbon nanotubes nanocomposite on bacillus subtilis biofilm development. Materials (Basel).

[14] Marchese A., Arciola C.R., Coppo E., Barbieri R., Barreca D., Chebaibi S., Sobarzo-Sánchez E., Nabavi S.F., Nabavi S.M., Daglia M. (2018). The natural plant compound carvacrol as an antimicrobial and anti-biofilm agent: mechanisms, synergies and bio-inspired anti-infective materials. Biofouling.

[15] Taglietti A., Dacarro G., Barbieri D., Cucca L., Grisoli P., Patrini M., Arciola C.R., Pallavicini P. (2019). High bactericidal self-assembled nano-monolayer of silver sulfadiazine on hydroxylated material Surfaces. Materials (Basel).

[16] Wu S., Li A., Zhao X., Zhang C., Yu B., Zhao N., Xu F.J. (2019). Silica-coated gold−silver nanocages as photothermal antibacterial agents for combined anti-infective therapy. ACS Appl. Mater. Interfaces.

[17] Shenashen M.A., El-Safty S.A., Elshehy E.A. (2014). Synthesis, morphological control, and properties of silver nanoparticles in potential applications. Part. Part. Syst..

[18] Burdusel A.C., Gherasim O., Grumezescu A.M., Mogoanta L., Ficai A., Andronescu E. (2018). Biomedical Applications of Silver Nanoparticles: An Up-to-Date Overview. Nanomaterials.

[19] Nakamura S., Sato M., Sato Y., Ando N., Takayama T., Fujita M., Ishihara M. (2019). Synthesis and application of silver nanoparticles (Ag NPs) for the prevention of infection in healthcare workers. Int. J. Mol. Sci..

[20] Sheehy K., Casey A., Murphy A., Chambers G. (2015). Antimicrobial properties of nano-silver: A cautionary approach to ionic interference. J. Colloid Interface Sci..

[21] Sportelli M.C., Picca R.A., Cioffi N., Phoenix D.A., Harris F., Dennison S.R. (2014). Nano-antimicrobials based on metals. Novel Antimicrobial Agents and Strategies.

[22] Oktar F.N., Yetmez M., Ficai D., Ficai A., Dumitru F., Pica A. (2015). Molecular mechanism and targets of the antimicrobial activity of metal nanoparticles. Curr. Top. Med. Chem..

[23] Pasquet J., Chevalier Y., Pelletier J., Couval E., Bouvier D., Bolzinger M.A. (2014). The contribution of zinc ions to the antimicrobial activity of zinc oxide. Colloids Surf. A Physicochem. Eng. Asp..

[24] Tarushi A., Psomas G., Raptopoulou C.P., Kessissoglou D.P. (2009). Zinc complexes of the antibacterial drug oxolinic acid: Structure and DNA-binding properties. J. Inorg. Biochem..

[25] Xu J., Ding G., Li J., Yang S., Fang B., Sun H., Zhou Y. (2010). Zinc-ion implanted and deposited titanium surfaces reduce adhesion of *Streptococcus mutans*. Appl. Surf. Sci..

[26] Nel A., Xia T., Madler L., Li N. (2006). Toxic potential of materials at the nanolevel. Science.

[27] Sportelli M.C., Picca R.A., Cioffi N. (2016). Recent advances in the synthesis and characterization of nano-antimicrobials. Trends Analyt. Chem..

[28] Vimbela G.V., Ngo S.M., Fraze C., Yang L., Stout D.A. (2017). Antibacterial properties and toxicity from metallic nanomaterials. Int. J. Nanomedicine..

[29] Queffélec C., Petit M., Janvier P., Knight D.A., Bujoli B. (2012). Surface modification using phosphonic acids and esters. Chem. Rev..

[30] Donnadio A., Nocchetti M., Costantino F., Taddei M., Casciola M., da Silva Lisboa F., Vivani R. (2014). A layered mixed zirconium phosphate/phosphonate with exposed carboxylic and phosphonic groups: X-ray powder structure and proton conductivity properties. Inorg. Chem..

[31] Costantino F., Vivani R., Bastianini M., Ortolani L., Piermatti O., Nocchetti M., Vaccaro L. (2015). Accessing stable zirconium carboxyaminophosphonate nanosheets as support for highly active Pd nanoparticles. Chem. Commun..

[32] Kozell V., Giannoni T., Nocchetti M., Vivani R., Piermatti O., Vaccaro L. (2017). Immobilized palladium nanoparticles on zirconium carboxy-aminophosphonatesnanosheets as an efficient recoverable heterogeneous catalyst for suzuki−miyaura and heck coupling. Catalysts.

[33] Costantino F., Nocchetti M., Bastianini M., Lavacchi A., Caporali M., Liguori F. (2018). Robust zirconium phosphate−phosphonate nanosheets containing palladium nanoparticles as efficient catalyst for alkynes and nitroarenes hydrogenation reactions. ACS Appl. Nano Mater..

[34] Shearan S.J., Stock N., Emmerling F., Demel J., Wright P.A., Demadis K.D., Vassaki M., Costantino F., Vivani R., Sallard S. (2019). New directions in metal phosphonate and phosphinate. Chem. Cryst..

[35] Alberti G., Costantino U., Alberti G., Bein T. (1996). Solid-State Supramolecular Chemistry: Two and Three-dimensional Inorganic Networks, of Comprehensive supramolecular Chemistry.

[36] Ambrogi V., Costantino U., Nocchetti M., Perioli L. (2008). Hydrotalcite-like compounds: Versatile layered hosts of molecular anions with biological activity. Micropor. Mesopor. Mater..

[37] Saxena V., Diaz A., Clearfield A., Batteas J.D., Delwar Hussain M. (2013). Zirconium phosphate nanoplatelets: A biocompatible nanomaterial for drug delivery to cancer. Nanoscale.

[38] Bastianini M., Costenaro D., Bisio C., Marchese L., Costantino U., Vivani R., Nocchetti M. (2012). On the intercalation of the iodine–iodide couple on layered double hydroxides with different particle sizes. Inorg. Chem..

[39] Heli B., Morales-Narváez E., Golmohammadi H., Ajji A., Merkoçi A. (2016). Modulation of population density and size of silver nanoparticles embedded in bacterial cellulose via ammonia exposure: Visual detection of volatile compounds in a piece of plasmonic nanopaper. Nanoscale.

[40] Fan W., Sun Q., Li Y., Tay F.R., Fan B. (2018). Synergistic mechanism of Ag(+)-Zn(2+) in anti-bacterial activity against *Enterococcus faecalis* and its application against dentin infection. J. Nanobiotechnol..

[41] Pajares-Chamorro N., Shook J., Hammer N.D., Chatzistavrou X. (2019). Resurrection of antibiotics that methicillin-resistant *Staphylococcus aureus* resists by silver-doped bioactive glass-ceramic microparticles. Acta Biomater..

